# Multi-omics analyses of red blood cell reveal antioxidation mechanisms associated with hemolytic toxicity of gossypol

**DOI:** 10.18632/oncotarget.21779

**Published:** 2017-10-10

**Authors:** Chaohua Tang, Qingshi Meng, Kai Zhang, Tengfei Zhan, Qingyu Zhao, Sheng Zhang, Junmin Zhang

**Affiliations:** ^1^ State Key Laboratory of Animal Nutrition, Institute of Animal Science, Chinese Academy of Agricultural Sciences, Beijing, China; ^2^ Scientific Observing and Experiment Station of Animal Genetic Resources and Nutrition in North China, Ministry of Agriculture, Beijing, China; ^3^ Institute of Biotechnology, Cornell University, Ithaca, NY, USA

**Keywords:** gossypol, red blood cell, hemolytic toxicity, metabolomics, proteomics

## Abstract

Gossypol is an antiproliferative drug with limited use due to its hemolytic toxicity. In this study, accelerated hemolysis was observed in the cows treated with gossypol. Comparative metabolomics were used to gain responsive pathways in the red blood cell (RBC) to the treatment, which were crossly validated by parallel iTRAQ-based proteomic analysis and enzyme activity assay. We found that gossypol treatment appeared to considerably activate pentose phosphate pathway (PPP) with an increased key product of ribose-5-phosphate and the increased abundance and activity of several key enzymes such as 6-phosphogluconate dehydrogenase, flavin reductase, and ribose-phosphate pyrophesphokinase. Meanwhile, a decreased glycolysis metabolism was observed, as many input metabolites of glycolysis were reduced in the gossypol group, whereas its distal metabolites were unchanged, along with decreased abundance of triosephosphate isomerase and increased abundance of enzymes catalyzing several distal glycolytic steps. Oxidative reduction pathways were also remarkably affected as we found a decreased substrate of flavin reductase, glutathione disulfide, increased glutathione reductase activity, and increased abundance and activity of glutathione S-transferase with the increase of its catalytic product, cysteine. Our results demonstrated that glycolysis, PPP, and oxidative reduction pathways of RBC were all involved in RBC’s response to the hemolytic toxicity of gossypol.

## INTRODUCTION

Gossypol is a polyphenolic compound derived from the cotton plant and has gained great interest due to its contraceptive activity. Besides its antifertility effects, other biological properties have also been reported [1, 2], of which antiproliferative effects have been recently drawn considerable attention [3, 4]. The use of gossypol as an alternative anticancer drug is limited by two main side effects, which include (a) the occurrence of hypokalaemic paralysis [5] and (b) the acceleration of eryptosis, which may trigger hemolysis and anemia [6]. The available evidence on animals and man suggests that the direct toxic effect of gossypol on the renal tubules leads to the renal leakage of potassium and results in hypokalaemic paralysis [5]. However, the mechanism of the hemolytic toxicity of gossypol *in vivo* has not been reported.

The most important task of red blood cell (RBC) is to bind and transport oxygen, which requires passage through microcapillaries. The latter is achieved by a drastic modification of its biconcave shape, made possible only by the loss of the nucleus and cytoplasmic organelles and, consequently, the lack of ability to synthesize proteins [7]. Yet, a number of vital pathways are still active in RBC, which contributed to generate energy and reducing power to perform its essential functions [8]. As soon as the RBC cannot fulfill its vital functions anymore, it will eventually lose its biconcave shape and eryptosis happens. Therefore, it is important to consider that RBC can accumulate modifications on metabolites and proteins that represent the long-term status of the body. However, the degree of hemolysis not only depends on the severity of RBC functions impairment, but also relies on the ability to compensate for the abnormalities by reticulocytes.

As a clinical sign of gossypol toxicity, the increases of RBC osmotic fragility induced by gossypol were reported extensively in animals [9, 10]. In recent years, an *in vitro* study showed that treatment of RBC with gossypol stimulates Ca^2+^ entry and induces eryptosis [6]. The subsequent increase in cytosolic Ca^2+^ leads to activation of Ca^2+^-sensitive K^+^ channels [11], resulting in K^+^ exit, hyperpolarization, and Cl^−^ exit [12, 13]. Cellular KCl loss, together with osmotically obliged water, results in cell-membrane scrambling and cell shrinkage [6], hallmarks of suicidal erythrocyte death. Despite cation-homeostasis dysregulation, other mechanisms might also contribute to the hemolytic toxicity of gossypol. These include (a) gossypol binds to iron [14] and, in so doing, produce a gossypol-iron complex that may cause iron deficiency, thereby affecting hemoglobin (HGB) generation and influencing RBC function; (b) gossypol possesses antioxidant and pro-oxidant properties [15] and could induce oxygen-radical formation [16], an indicator of oxidative stress, which could trigger eryptosis [17]. Nevertheless, besides its cation-homeostasis dysregulation found *in vitro* [6], little is known about the gossypol effects *in vivo*. In this context, metabolomics and proteomics are the methods of choice to investigate the RBC’s response to the hemolytic toxicity of gossypol at the molecular level, given the fact that the mature RBCs do not contain RNA or DNA [18–21].

In this study, an increased hemolysis was observed after gossypol treatment of dairy cows. Metabolomics study showed that glycolysis, pentose phosphate pathway, and oxidative reduction pathways were profoundly affected. A parallel proteomics analysis, combined with targeted-based enzyme assay, found that the abundance and activity change of most enzymes involved in the above pathways were in good agreement with the alteration of metabolome. Here, we reported multi-omics analysis results, which provided not only new insights into the molecular mechanisms on the RBC in response to the hemolytic toxicity of gossypol and but also shed light on the continuing studies of gossypol as a potential anticancer drug.

## RESULTS

### Gossypol administration induces RBC hemolysis

**Figure 1:**
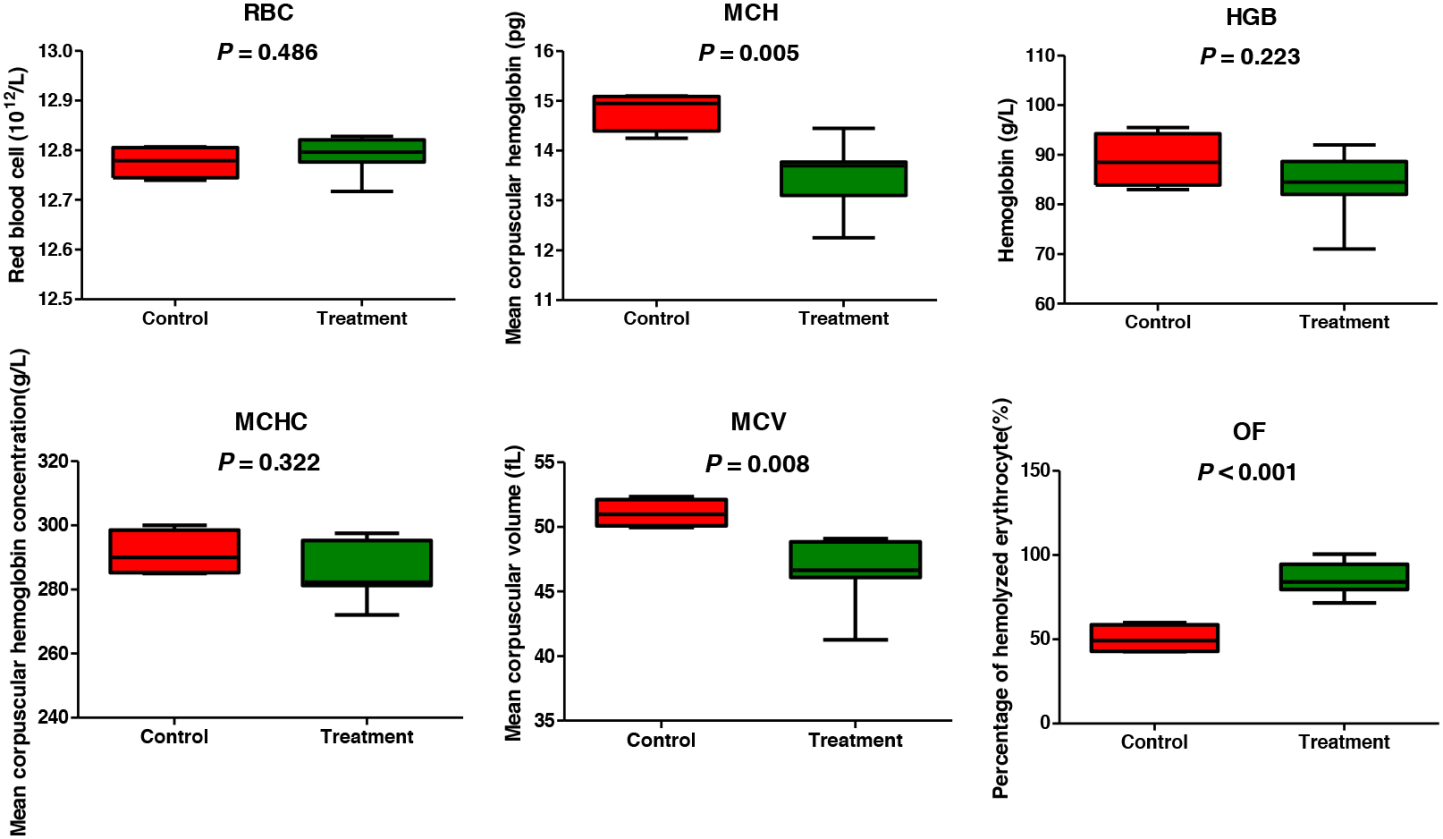
Red blood cell parameters and osmotic fragility of dairy cows from the control and gossypol-treated groups

RBC parameters and percentage of hemolytic RBC in 0.8% NaCl-phosphate buffer are presented in Figure 1. RBC, HGB, and MCHC values between the control and gossypol-treated groups were not significantly different. MCH and MCV were significantly decreased by gossypol treatment (*p* = 0.005 and 0.008, respectively), and the percentage of hemolytic RBC in 0.8% NaCl-phosphate buffer in the gossypol-treated group was 86.08%, which was significantly higher than that observed in the control group (50.28%; *p* = 0.00007).

### Quality control of metabolomics and proteomics analysis

**Figure 2:**
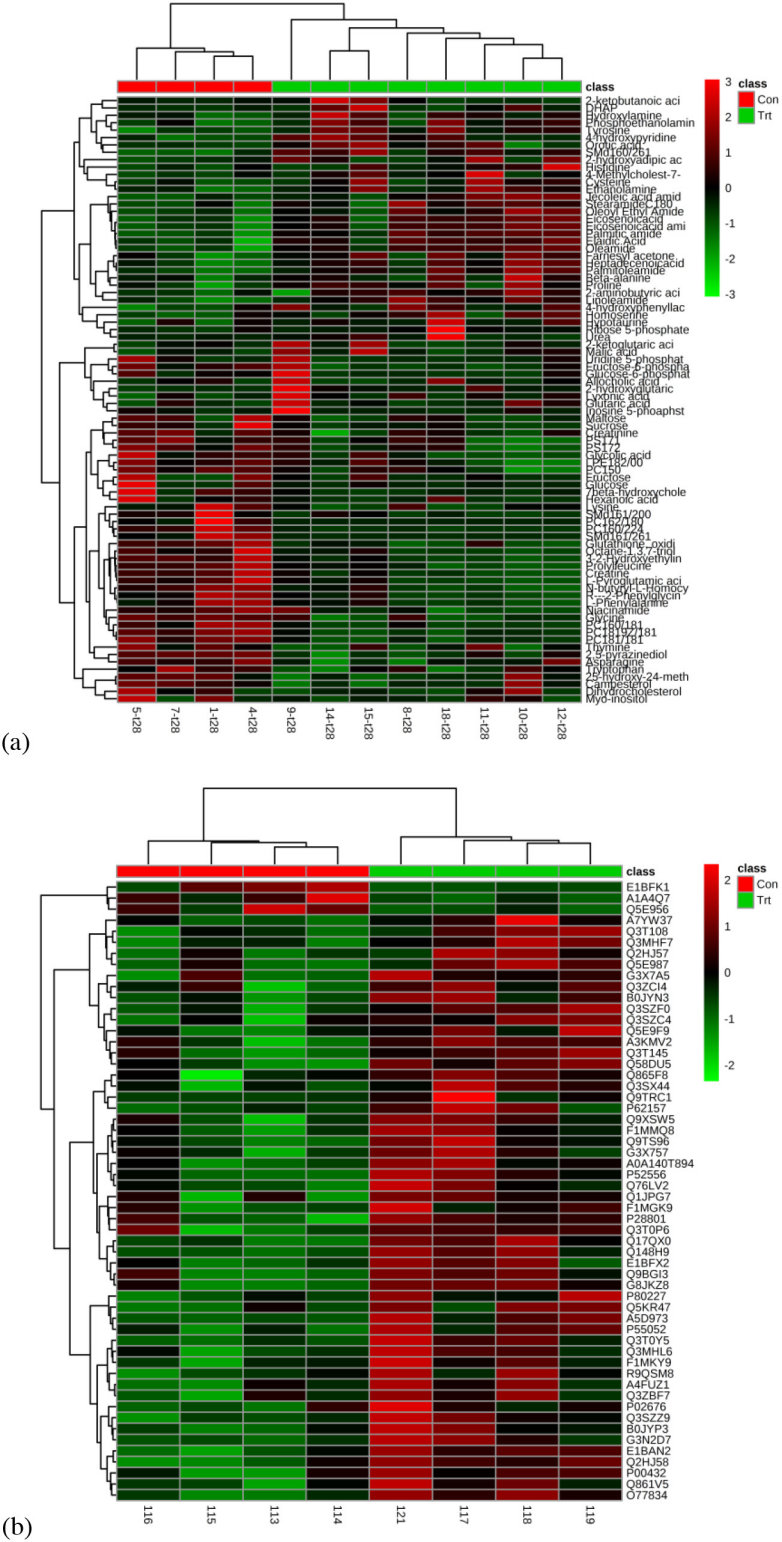
Heat map of all differential metabolites **(a)** and proteins **(b)** in red blood cells of cows from control and gossypol-treated groups.

The data quality from GC-MS metabolomic analysis was evaluated according to the intesities of internal standards added during sample preparation. The relative standard derivations (RSDs) for the internal standards 13C3-15N-L-alanine, 13C5-15N-L-valine, 13C6-15N-L-leucine, and 13C6-15N-L-isoleucine were 5.08%, 4.59%, 4.82%, and 3.52%, respectively. Additionally, the data quality from GC-MS and UPLC-QTOF metabolomics analysis was assessed by three quality control samples analyzed at the beginning, middle, and end of data acquisition. The RSDs for 91.78% and 72.54% features were < 30% in GC-MS and UPLC-QTOF metabolomic data sets, respectively. Proteomic data were assessed for the variation of identified and quantified proteins, which shown that the RSDs for 74.05% and 77.29% of the quantified proteins were < 30% in the control and gossypol-treated groups, respectively. Hierarchical cluster analysis of the differential metabolites and proteins displayed as heat maps clearly shows that samples were clustered into two groups (Figure 2). All these results suggested reproducible technical replicates and acceptable biological variation in this study.

### Altered RBC metabolome by gossypol treatment

**Figure 3:**
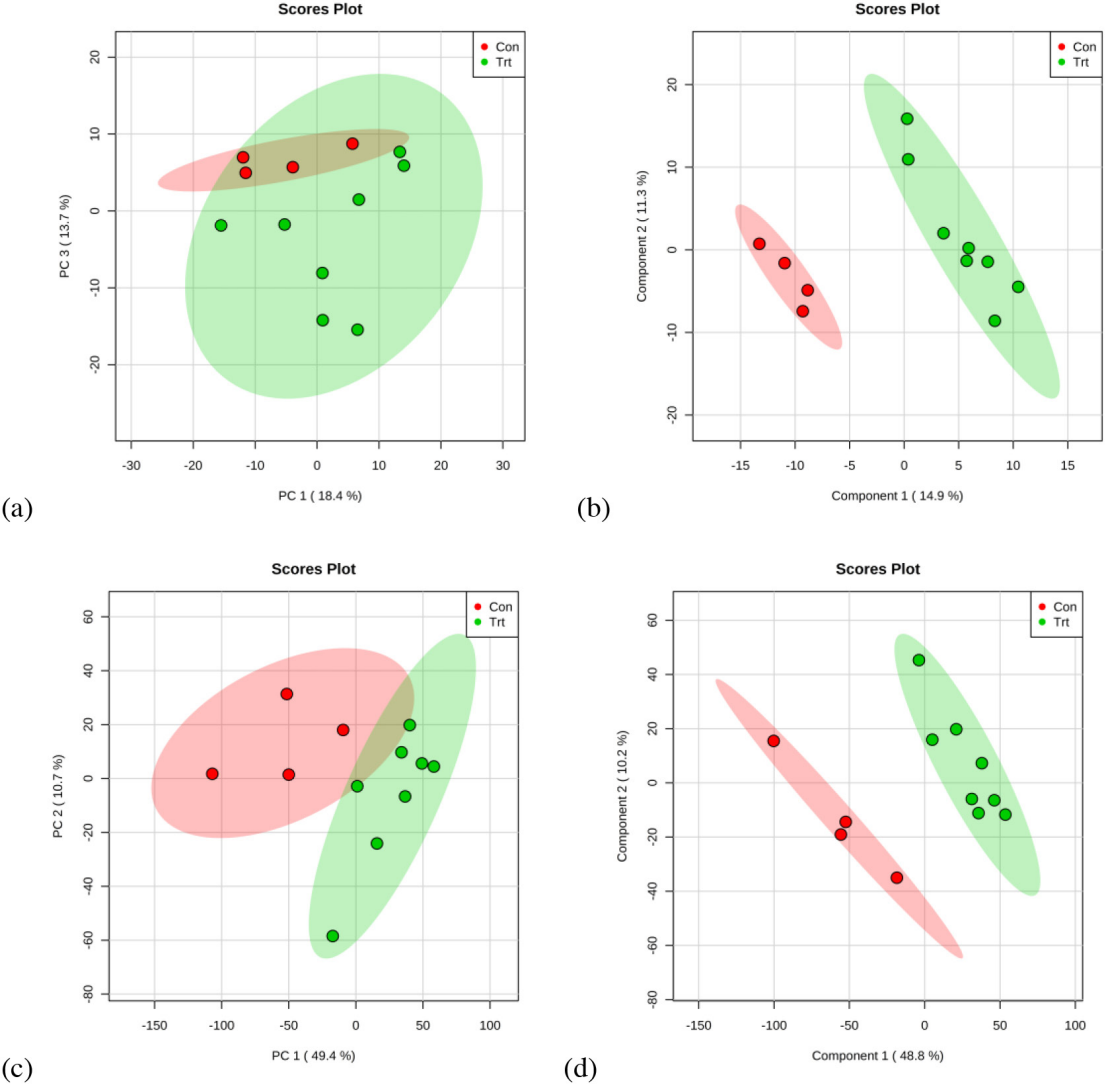
PCA and PLS-DA based on data derived from GC-MS **(a, b)** and UPLC-QTOF **(c, d)** metabolomics analysis of red blood cell from dairy cows from the control and gossypol-treated groups.

Multivariate statistical analysis was used to detect metabolic differences in the GC-MS and UPLC-QTOF data sets between the control and gossypol groups (Figures 3). For GC-MS data, four principal components were calculated in PCA analysis, with an R2X value of 0.605. There was a tendency of cluster between the control and gossypol-treated groups in the score plot of PCA, and the gossypol-treated group was well discriminated from the control group according to PLS-DA (Figure 3). Three principal components were calculated to build the PLS-DA model with the following parameters: R2X = 0.414, R2Y = 0.996, and Q2 = 0.646. The differential metabolites between the two groups were selected based on the *p-*values (*p* < 0.05) and the FC (FC > 1.4 or < 0.67). A total of 30 metabolites meeting any of the above criteria and being chosen for pathway analysis are shown in Table 1 and source dataset are presented in Supplementary Material 1.

**Table 1:** Differential metabolites screen for pathway analysis identified by GC-MS in red blood cells of cows from the control and gossypol-treated groups

No.	m/z	Rt (min)	*p-*value	FC	Compounds
***p*-value < 0.05**					
1	205	7.56	0.031	0.839	Glycolic acid
2	102	8.5	0.004	0.568	Glycine
3	152	9.17	0.017	1.153	4-hydroxypyridine
4	174	11.56	0.033	1.260	Ethanolamine
5	241	13.58	< 0.001	0.571	2,5-pyrazinediol
6	243	16.56	0.034	0.808	Asparagine
7	142	18.06	0.029	1.549	Proline
8	188	21.02	0.038	1.484	Phosphoethanolamine
9	179	22.28	0.006	1.397	Tyrosine
10	179	22.41	0.047	1.711	4-hydroxyphenyllactic acid
11	311	23.78	0.015	0.508	Palmitoleic acid
12	202	25.53	0.047	0.899	Tryptophan
13	456	32.04	0.016	0.249	7beta-hydroxycholesterol
14	386	32.19	0.049	0.660	25-hydroxy-24-methylcholesterol
15	343	32.27	0.050	0.666	Campesterol
**FC > 1.4 or < 0.67**					
1	173	7.54	0.114	0.596	Hexanoic acid
2	261	14.59	0.303	1.403	Glutaric acid
3	218	15.5	0.086	1.449	Homoserine
4	220	17.6	0.083	1.786	Cysteine
5	129	18	0.158	3.940	2-hydroxyglutaric acid
6	198	18.01	0.425	1.712	2-ketoglutaric acid
7	129	19.53	0.137	1.453	2-hydroxyadipic acid
8	315	20.68	0.155	3.470	Dihydroxy acetone phosphate
9	103	22.09	0.267	0.569	Fructose
10	319	22.58	0.103	0.296	Glucose
11	291	23.41	0.636	3.377	Pantothenic acid
12	217	24.38	0.148	0.636	Myo-inositol
13	315	24.53	0.321	1.699	Ribose 5-phosphate
14	361	28.5	0.062	0.450	Maltose
15	269	32.7	0.147	2.046	4-Methylcholest-7-en-3-ol

The *p*-value was calculated from Student’s *t* test. FC (fold change), defined as normalized peak area of metabolites in treatment group/control group.

The dataset derived from UPLC-QTOF analysis was examined similar to that described for the GC-MS dataset (Figure 3). Two principal components were calculated in the PCA, with R2X = 0.531 and the control and gossypol groups clustered into two groups. The two groups were well discriminated according to the supervised methods PLS-DA. Three components were obtained in the PLS-DA, with R2X = 0.579, R2Y = 0.999, and Q2 = 0.874. Differential metabolites between the two groups were selected based on the criteria stated in the description of GC-MS data analysis. A total of 36 metabolites meet all the criteria were found, with the abundance of 13 being elevated and 23 decreased in the gossypol-treated group as compared with the control group (Table 2). The 36 metabolites were classified into fatty amides (nine metabolites), phosphosphingolipids (three metabolites), glycerophosphoserines (two metabolites), glycerophosphocholines (six metabolites), and others (16 metabolites). The source dataset are presented in the Supplementary Material 2.

**Table 2:** Differential metabolites identified by UPLC-QTOF in red blood cells of cows from the control and gossypol-treated groups

No.	Rt (min)	m/z	*p*-value	FC	Compounds
1	15.84	619.6111	0.029	2.854	Oleoyl Ethyl Amide
2	13.24	254.249	0.006	2.466	Palmitoleamide
3	14.67	308.2958	0.031	1.916	Jecoleic acid amide
4	15.49	284.2961	0.035	1.904	Stearamide (C18:0)
5	13.57	263.2381	0.003	1.895	Linoleamide
6	17.66	815.696	0.004	1.848	SM (d16:0/26:1)
7	13.69	268.2636	0.009	1.821	Heptadecenoic acid amide
8	15.55	310.3122	0.005	1.740	Eicosenoic acid amide
9	13.41	263.2381	0.024	1.696	Farnesyl acetone
10	14.06	256.2659	0.001	1.673	Palmitic amide
11	15.55	293.2841	0.008	1.558	Eicosenoic acid
12	14.41	282.2813	0.001	1.404	Oleamide
13	14.41	265.2529	0.003	1.253	Elaidic Acid
14	11.34	496.3402	0.019	0.673	PC(15:0)
15	10.79	478.2933	0.047	0.662	LPE(18:2/0:0)
16	12.1	524.2974	0.050	0.662	PS(17:1)
17	0.96	114.0674	0.004	0.637	Creatinine
18	0.79	130.0865	0.003	0.633	L-Pyroglutamic acid
19	11.05	522.282	0.017	0.628	PS(17:2)
20	3.77	229.1559	0.001	0.617	Prolylleucine
21	4.2	166.0874	0.011	0.606	L-Phenylalanine
22	4.2	120.0819	0.008	0.588	(R)-(-)-2-Phenylglycinol
23	1.02	132.0779	0.001	0.586	Creatine
24	4.92	188.0722	0.001	0.586	N-butyryl-L-Homocysteine thiolactone
25	6.67	163.134	0.004	0.503	Octane-1,3,7-triol
26	1.78	123.0563	0.019	0.485	Niacinamide
27	3.58	613.1584	0.001	0.433	Glutathione, oxidized
28	14.16	144.0812	< 0.001	0.390	3-(2-Hydroxyethyl) indole
29	0.98	365.1049	0.042	0.357	Sucrose
30	17.66	731.6012	0.022	0.231	SM (d16:1/20:0)
31	17.65	786.5994	< 0.001	0.197	PC(18:1/18:1)
32	14.25	786.5963	< 0.001	0.188	PC(18:1(9Z)/18:1)
33	17.64	810.597	< 0.001	0.159	PC(16:0/22:4)
34	17.65	760.5834	0.001	0.135	PC(16:0/18:1)
35	13.58	813.6794	0.006	0.043	SM (d16:1/26:1)
36	17.65	758.5666	0.034	0.015	PC(16:2/18:0)

The *p*-value was calculated from Student’s *t* test. FC (fold change), defined as normalized peak area of metabolites in treatment group/control group.

### Cross-validation of the metabolomics results with proteomics analysis

To verify the metabolomics results, proteomics analysis was conducted to study the abundance change of the enzymes involved in the pathways of interest. A summary of the proteomics analysis results is presented in Supplementary Material 3. A total of 185 proteins were identified, of which 56 sequences were differentially expressed between the two groups with the criteria *p*-value of 0.1. Then, these proteins were categorized based on their biological process and summarized in Table 3. Most enzymes involved in glycolysis, pentose phosphate pathway (PPP), and oxidative reduction were increased in abundance, except for triosephosphate isomerase 1, which catalyzes the transformation between glyceraldehydes-3-phosphate and dihydroxyacetone phosphate, was decreased in abundance. Some other proteins including: membrane proteins, proteasomes, anion exchange protein, calmodulin, heat shock protein were also increased in abundance by gossypol treatment.

**Table 3:** Primary functional classification on differentially expressed proteins by iTRAQ

Protein name	Accession	Fold change^a^	Trends ^b^	*p*-value
**Glycolysis**				
Katanin p60 ATPase-containing subunit A-like 2	E1BAN2	1.329 + 0.059	+	0.005
L-lactate dehydrogenase B	B0JYN3	1.390 + 0.152	+	0.021
Phosphoglycerate kinase	Q58DJ6	1.307 + 0.023	+	0.079
Pyruvate kinase 1	Q1JPG7	1.250 + 0.069	+	0.083
Triosephosphate isomerase	Q5E956	0.639 + 0.052	-	0.028
**Pentose phosphate pathway**				
6-phosphogluconate dehydrogenase	Q3ZCI4	1.420 + 0.143	+	0.087
Flavin reductase (NADPH)	P52556	1.215 + 0.077	+	0.012
Ribose-phosphate pyrophosphokinase 1	Q2HJ58	1.343 + 0.063	+	0.007
**Oxidative reduction**				
Catalase	P00432	1.183 + 0.051	+	0.032
Glutathione S-transferase P	P28801	1.472 + 0.118	+	0.051
Lactoylglutathione lyase	A4FUZ1	1.252 + 0.130	+	0.063
Peroxiredoxin-2	Q9BGI3	1.292 + 0.088	+	0.038
Peroxiredoxin-6	O77834	1.483 + 0.124	+	0.004
Superoxide dismutase [Cu-Zn]	F1MNQ4	1.392 + 0.173	+	0.024
Thioredoxin	G8JKZ8	1.503 + 0.097	+	0.009
**Others**				
26S protease regulatory subunit 7	Q5E9F9	1.230 + 0.112	+	0.045
Alpha-hemoglobin-stabilizing protein	Q865F8	1.321 + 0.076	+	0.099
Hemoglobin, theta 1	A1A4Q7	0.467 + 0.074	-	0.017
Anion exchange protein	Q9XSW5	1.203 + 0.074	+	0.052
Calmodulin	P62157	1.669 + 0.389	+	0.057
Erythrocyte membrane protein band 4.2	B0JYP3	1.212 + 0.104	+	0.036
Tropomyosin alpha-3 chain	Q5KR47	1.169 + 0.082	+	0.050
14-3-3 protein beta/alpha	A0A140T894	1.238 + 0.095	+	0.025
Acylamino-acid-releasing enzyme	P80227	1.324 + 0.165	+	0.038
Acyl-CoA-binding domain-containing protein 7	Q3SZF0	1.312 + 0.083	+	0.012
Serine/threonine-protein phosphatase 2A 65 kDa regulatory subunit A alpha isoform	Q32PI5	1.459 + 0.171	+	0.006
Alpha-2-macroglobulin	R9QSM8	1.673 + 0.372	+	0.062
ANTIGONDADOTROPIC DECAPEPTIDE, AGD	Q9TRC1	3.996 + 2.332	+	0.028
CD58 protein	A7YW37	1.806 + 0.454	+	0.030
Coactosin-like protein	Q2HJ57	1.282 + 0.162	+	0.076
Complement C3	G3X7A5	1.751 + 0.295	+	0.050
Fatty acid-binding protein, epidermal	P55052	1.196 + 0.078	+	0.026
FGG protein/Fibrinogen gamma-B chain	P12799	1.708 + 0.287	+	0.016
Fibrinogen beta chain	P02676	1.666 + 0.446	+	0.087
Heat shock protein HSP 90-alpha	Q76LV2	1.194 + 0.104	+	0.048
Malate dehydrogenase, cytoplasmic	Q3T145	1.367 + 0.095	+	0.020
N(G), N(G)-dimethylarginine dimethylaminohydrolase 2	Q3SX44	1.207 + 0.081	+	0.041
NSFL1 cofactor p47	Q3SZC4	1.232 + 0.068	+	0.048
Nudix (Nucleoside diphosphate linked moiety X)-type motif 5	Q17QX0	1.325 + 0.090	+	0.003
Peptidyl-prolyl cis-trans isomerase	Q861V5	1.253 + 0.123	+	0.051
Prostaglandin E synthase 3	Q3ZBF7	1.177 + 0.090	+	0.079
Proteasome subunit alpha type-2	Q3T0Y5	1.521 + 0.214	+	0.010
Proteasome subunit alpha type-3	Q58DU5	1.266 + 0.050	+	0.008
Proteasome subunit alpha type-5	Q5E987	1.228 + 0.097	+	0.035
Proteasome subunit beta type-4	Q3T108	1.368 + 0.160	+	0.036
Nucleolar protein NOP52	A1L546	1.807 + 0.271	+	0.003
S-methyl-5'-thioadenosine phosphorylase	Q3MHF7	1.384 + 0.137	+	0.016
Transitional endoplasmic reticulum ATPase	G3X757	1.114 + 0.079	+	0.092
TSC22 domain family protein 1	Q3MHL6	1.407 + 0.186	+	0.047
UV excision repair protein RAD23 homolog A	A3KMV2	1.235 + 0.042	+	0.028

^a^ Fold change: the ratios for the tags in the gossypol-treated group/tags in the control group.

^b^ +: increased abundance; -: decreased abundance.

### Confirmation of proteomics analysis by enzyme activity assay

**Figure 4:**
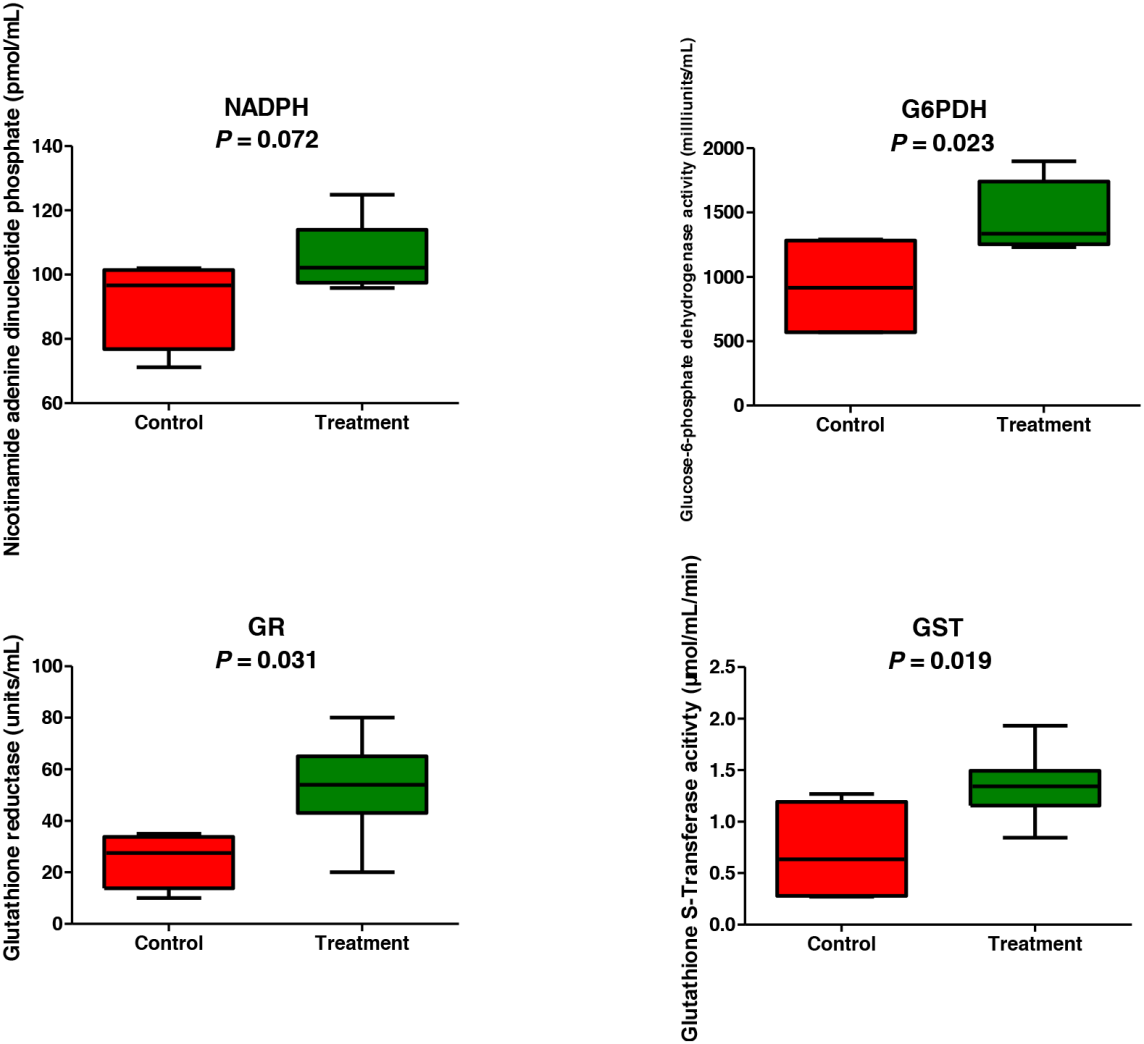
Confirmation of metabolomics and proteomics results by assaying oxidation-reduction enzymes activities

Because both the metabolomics and proteomics results consistently indicated that the oxidation-reduction is one of the most responsive pathways to the gossypol treatment, activity assays were performed for 6-phosphogluconate dehydrogenase, NADHP, glutathione S-transferase, and glutathione reductase. As shown in Figure 4, the activities of 6-phosphogluconate dehydrogenase, glutathione S-transferase, and glutathione reductase in the gossypol-treated group were all higher than those in the control group (*p* = 0.023, 0.019 and 0.031, respectively). We also measured the concentrations of NADPH using commercial kits and found that the levels of NADPH in the gossypol-treated group were increased (*p* = 0.072). These results showed a strong correlation with the proteomics data.

### Integrating metabolomics and proteomics pathway analysis

**Figure 5:**
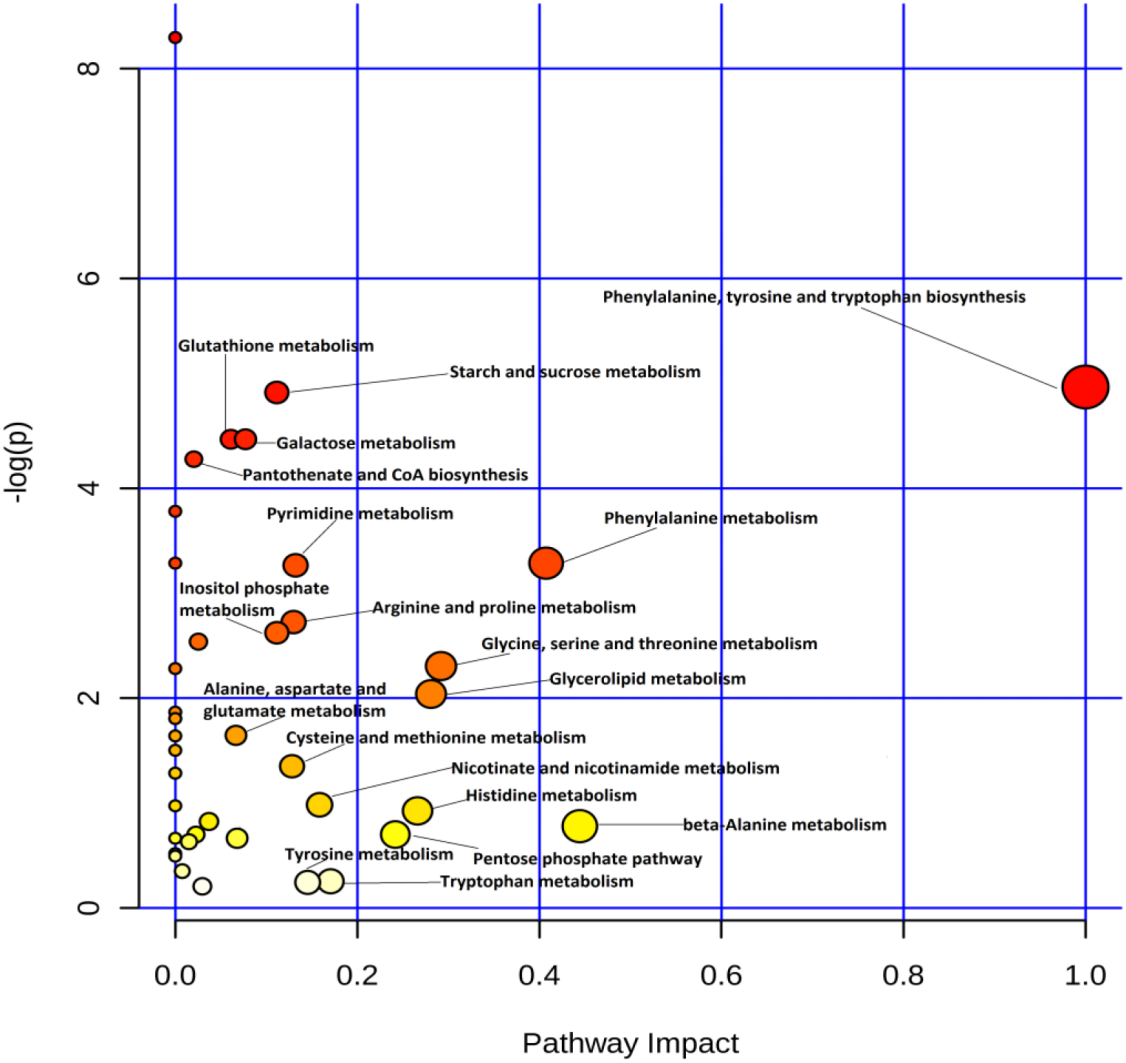
Pathway analysis of differential metabolites identified in red blood cells of cows from the control and gossypol-treated groups using MetaboAnalyst 3.0

**Figure 6:**
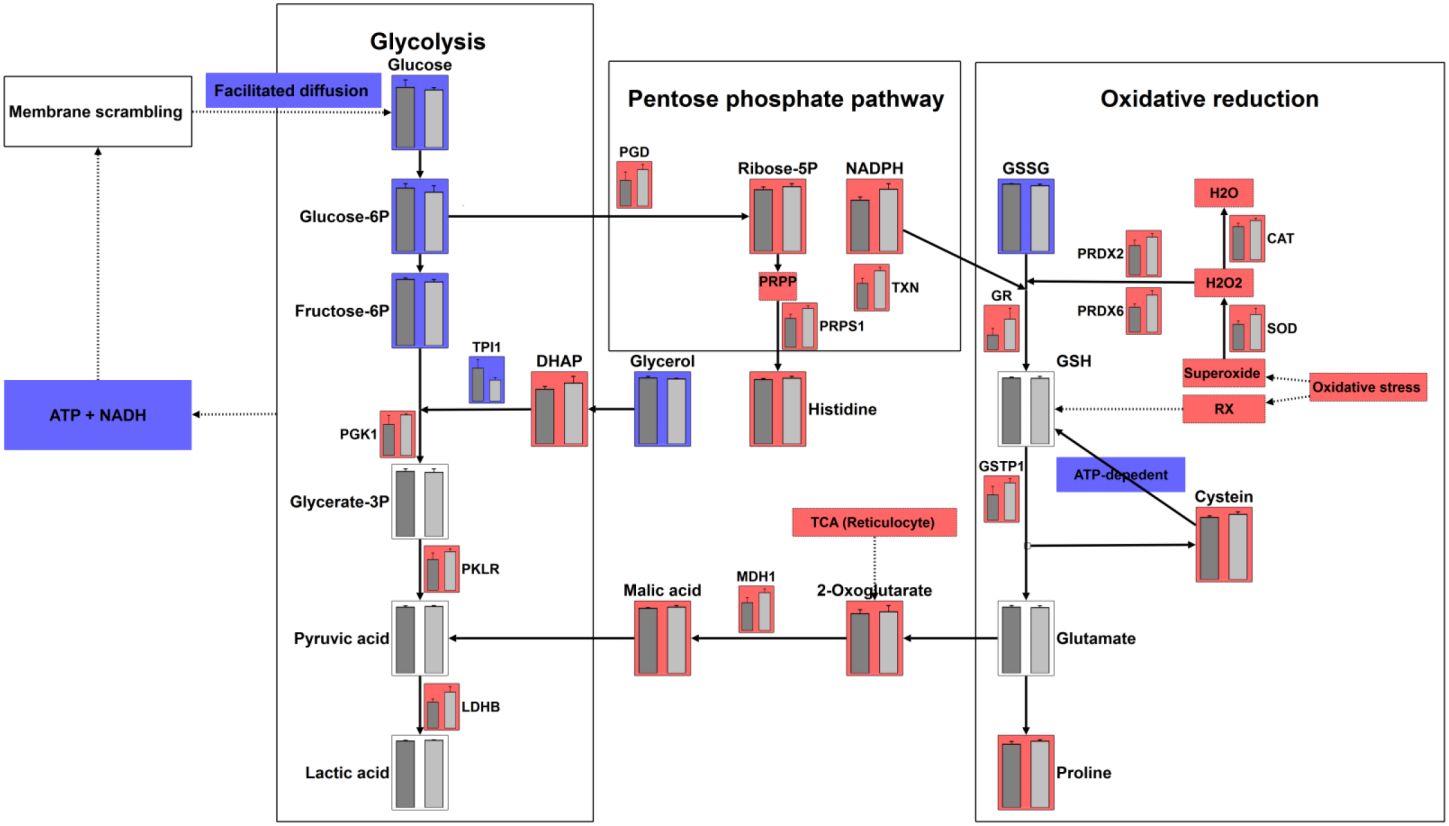
Integrating metabolomics and proteomics pathways: glycolysis, pentose phosphate pathway, oxidative reduction pathways Red colored bar represents an increased abundance of metabolites or enzymes in the gossypol-treated group. Blue colored bar represents a decreased abundance of metabolites or enzymes in the gossypol-treated group.

Differential metabolites were chosen for pathway analysis using MetaboAnalyst 3.0 [22] and grouped manually (Figures 5 and 6). It’s showed that energy metabolism (glycolysis and the PPP), glutathione metabolism, and amino acid metabolism, among others were most profoundly affected by gossypol treatment. Glycolysis input compounds were reduced based on our detection of decreased glucose and its intermediate products glucose-6-phospahte (FC = 0.778) and fructose-6-phosphate (FC = 0.700). While the distal glycolysis products including pyruvate and lactate showed no changes. PPP was activated based on evidence of the accumulation of its end product, ribose-5-phosphate. We also observed decreases in glutathione disulfide levels, and the increased catabolites of glutathione, cysteine. The differential metabolites identified by UPLC-QTOF also included phospholipids. Because mature RBC contains no cellular organelles and cannot utilize fatty acids to synthesize lipids, we suspect that these differential phosphatidycholine, phosphatidylserine, and sphingomyelin are likely from either the cell membrane, or the reticulocyte.

We further mapped the enzymes involved in the three pathways of interest (Figure 6). The abundance of glycolytic enzymes including enolase 1, phosphofructokinase, glyceraldehydes-3-phosphate dehydrogenase, and phosphoglycerate mutase 2 were not affected by gossypol. Triosephosphate isomerase 1 contents however, were decreased in the gossypol-treated group and this may cause the accumulation of dihydroxy acetone phosphate. In addition, L-lactate dehydrogenase, phosphoglycerate kinase, and pyruvate kinase 1 were elevated in the gossypol group, this may contribute to the unchanged levels of products in the distal of glycolysis, whereas the glycolysis input was decreased. Enzyme catalyzing the glucose-6-phosphate in the entrance step of the PPP, 6-phosphogluconate dehydrogenase, was up-regulated and increased activity, and this was in good agreement with the increases of the end products of PPP, reducing power NADPH and ribose-5-phosphate. The elevated NADPH level was along with the increased glutathione reductase activity, which cooperated with each other to reduce glutathione disulfide to glutathione, and this was in line with the decreases of the glutathione disulfide. The glutathione S-transferase P was up-regulated and increased activity in the gossypol group, which may result in the increases of its catalysate, cysteine. Other oxidative reduction enzymes including, catalase, peroxiredoxin-2/6, thioredoxin and superoxide dismutase were all up-regulated, indicating an enhanced ability of antioxidation to combat the increased oxidative stress induced by gossypol treatment.

## DISCUSSION

This study provided new insight into the RBC state at the molecular level in response to the hemolytic toxicity of gossypol through multi-omics analysis. Our hypothesis was formulated based on previous *in vitro* evidence showing that gossypol treatment induced suicidal erythrocyte death by stimulating Ca^2+^ entry [6]. The mechanism on Ca^2+^ triggering cell death includes (a) activation of Ca^2+^-sensitive K^+^ channels [11], the opening of K^+^ channels results in K^+^ exit, leading to cell membrane hyperpolarization, Cl^−^ exit, and loss of cellular KCl with osmotically obliged water [12]; (b) leads to cell-membrane scrambling, with subsequent phosphatidylserine exposure at the erythrocyte surface [23]. To complement with previous *in vitro* data elucidating gossypol-induced eryptosis based on cation-homeostasis dysregulation [6], we used both metabolomics and proteomics approaches in gaining additional pieces of evidence for the mechanisms on hemolytic toxicity of gossypol in RBC *in vivo*. In our study, increased hemolysis was observed, as the MCH and MCV were decreased and osmotic fragility was increased in the gossypol-treated group. This initial observation inspired us to take a global metabolomics approach on RBCs to screen changes of relevantly responsive pathways. We found that the most changed pathways to gossypol treatment were three associated pathways including glycolysis, PPP, and oxidative reduction pathways. Consistent results were also obtained in our proteomics analysis and targeted-based enzymes activities assay, and both metabolites and protein data sets indicated that there was a good causal correlation between the abundance of enzymes involved in the pathways and the alteration of metabolome, although the expression of some glycolytic enzymes seems regulated by dynamic and complex mechanisms.

Gossypol binds to mineral elements, and iron was demonstrated to be the most efficient antidote to gossypol toxicity [14]. Thus, gossypol traps iron, a key player in hemoglobin generation, and may explain the decreased MCH in the gossypol-treated group. MCV and osmotic fragility are indicators of eryptosis and hemolysis. The decreased MCV and increasing osmotic fragility observed here agreed well with previous studies reporting that treatment of gossypol resulted in RBC membrane scrambling and cell shrinkage *in vitro* [6] and increased osmotic fragility *in vivo* [9, 10]. Additionally, our results support the notion that eryptosis and hemolysis were accelerated *in vivo* by gossypol treatment.

The differential metabolites identified from both GC-MS and UPLC-QTOF were analyzed to determine their relevant pathways that were subsequently grouped manually. Lower levels of glucose and its intermediate products, including glucose-6-phosphate and fructose-6-phosphate, were initially observed in the gossypol-treated group. Glucose is the main energy source in RBC and enters RBC from circulating blood through facilitated diffusion. The disruption of the RBC membrane as a result of gossypol treatment was previously reported [6]; therefore, our observation of altered intracellular glucose levels agrees with those findings, although more evidence is needed to clarify the exact mechanisms involved. One of the most important biological roles of glycolysis is the production of ATP under anaerobic respiration, which is mainly used to maintain cation homeostasis. Energy depletion was also reported a stimulator of eryptosis [24]. Therefore, the reduction of glycolysis input may in turn exacerbate eryptosis and increase hemolysis. Furthermore, the regulation of glycolysis pathway was independently confirmed by proteomics results. A total of 8 glycolytic enzymes were identified, of which, the abundance of four, including phosphofructokinase, glyceraldeydes-3-phosphate dehydrogenase, phosphoglycerate mutase 2, and enolase 1 were not changed, whereas three of them, including pyruvate kinase 1, phosphoglycerate kinase, and L-lactate dehydrogenase were increased in abundance and one enzyme, triosephosphate isomerase 1 was decreased in abundance. The expression of glycolytic enzymes was partly in line with the metabolites alteration including accumulated dihydroxy acetone phosphate and unchanged glycerate-3-phosphate, pyruvic acid, and lactic acid levels, decreased input metabolites of glycolysis. First of all, the low level of input metabolites of glycolysis observed in this study is consistent with the elevated PPP pathway evidenced with an increased key PPP metabolomic product and some key enzymes involved in PPP pathway. Secondly, since some key metabolic enzymes (e.g. pyruvate kinase) were allosterically regulated by the relevant metabolites through either feedforward stimulation or feedback inhibition, we suspect that the increased expression of three enzymes in distal steps of glycolysis may be the result of a compensation effect to the decreasing of glycolysis input and potential low concentration of ATP which would allow for maintenance of normal level of the metabolites and ATP in those distal steps. This was consistent with what we found in metabolomics data with unchanged metabolites in the distal steps of glycolysis. It should be pointed out that as mature RBCs lose capability of protein synthesis, we anticipate that reticulocytes may play a role in the compensation process, which was documented in other RBC disease [7]. A physiological response to compensate for anemia caused by hemolysis is increased reticulocyte production. Reticulocytes still preserve cytoplasmic organelles and are thus capable of protein synthesis. Several enzymes, including hexokinase, pyruvate kinase, and aldolase, display much higher activity in reticulocytes and are often referred to as the age-related enzymes [25]. Moreover, the post-translational modifications of the glycolytic enzymes may play a role in the RBCs’ response to gossypol-induced oxidative stress, as it has been previously shown that activation of the PPP in RBCs by oxidative stress is at least in part mediated by reversible/irreversible oxidation of functional cysteine and histidine of glyceraldehyde 3-phosphate dehydrogenase [26].

Despite energy metabolism, RBCs also need reducing status generated from glycolysis and PPP to effectively perform its normal function. One type of reducing source is through the PPP, wherein NADP+ is converted into NADPH. This NADPH is used in the glutathione metabolism pathway to reduce glutathione disulfide to glutathione, which is the major reducing agent for RBC [7]. In this study, the products of PPP, ribose-5-phosphate and NADPH both were increased in the gossypol-treated group as detected by the metabolomics, proteomics, and targeted-based assay. This result indicated that more glucose entered into the PPP in response to gossypol treatment. Ratio of glycolysis/PPP flux constitutes an indirect parameter used to assess increased oxidative stress in RBC. Under normal steady state conditions, 92% of glucose is metabolized via glycolysis and 8% via the PPP [27]. Oxidation conditions will result in additional glucose molecules being metabolized through the PPP, which decreases the ratio of glycolysis/PPP flux [27, 28]. Thus, the decreased glycolysis input and the activation of the PPP may imply gossypol-induced oxidative stress, a property that was well documented before, which showed that gossypol exhibited pro-oxidant properties according to *in vitro* and *in vivo* studies based on elevations in reactive oxygen species with increased malondialdehyde production, and decreased glutathione concentrations [15, 16, 29, 30]. Further studies performing tracing experiments by incubating RBCs with 13C1, 2 or 13C1, 2, 3-glucose to confirm the activation of glycolysis vs PPP as observed in this study would be of great value. Along with the elevated NADPH, the glutathione reductase activity was increased, this could reduce more glutathione disulfide to glutathione and evidenced by lower level of the glutathione disulfide in the gossypol group. The enhanced transformation from glutathione disulfide to glutathione indicated an increased protective effect of glutathione in response to gossypol-induced stress, and was also confirmed by the increased abundance of glutathione S-transferase P in our proteomics data and the increased activity of glutathione S-tranferase in targeted-based assay, which catalyzes the conjugation of glutathione to a wide number of hydrophobic electrophiles. As glutathione transferase was up-regulated and activity increased, we also detected accumulation of the catabolites of glutathione, cysteine. Additionally, the increased cysteine may also result from the depression of *de novo* synthesis of glutathione, which is ATP dependent, as we demonstrated the decreases of glycolysis input. In spite of glutathione metabolism, other oxidative reduction enzymes, including superoxide dismutase, catalase, peroxiredoxin-2, peroxiredoxin-6, and thioredoxin were up-regulated in response to gossypol treatment. However, these complements did not appear sufficient to effectively protect RBC from oxidative stress, whereas the MCV and osmotic fragility were still significantly altered as shown at the beginning of this study.

Besides metabolites and enzymes involved in glycolysis, PPP, and oxidative reduction pathways, 2-oxoglutarate and malic acid were also identified in this study and in elsewhere [19], which may explained by transaminase activity and cytosolic malate dehydrogenase activity [31] or may come from the reticulocytes. Both 2-oxoglutarate and malic acid (FC = 1.286, *p* = 0.168) levels were increased in response to gossypol treatment, along with the increased abundance of the malate dehydrogenase. This may imply an enhanced TCA in the reticulocytes to supply more ATP to combat decreased ATP generation from glycolysis in mature RBC. The differential metabolites identified also included lipids; however, lipid synthesis is active in reticulocytes and suppressed in mature RBC [32]. Therefore, the differential lipids identified in this study may belong to the RBC membrane or from reticulocytes. The alteration of lipid concentrations in the gossypol-treated group may be an indicator that the RBC membrane was disrupted as a result of gossypol treatment. The reduction in six phosphatidycholine species, two phosphatidylserine species, and two sphingomyelin species in the gossypol-treated group may have indicated the decreased membrane fluidity, resulting in an increased osmotic fragility attributed to hemolysis.

In conclusion, we characterized the RBC state at molecular level in response to the hemolytic toxicity of gossypol by performing multi-omics analysis. Acceleration of hemolysis was observed after gossypol treatment and subsequent altered pathways were identified by untargeted metabolomics and further cross-verified by comparative proteomics, which later validated by targeted-based enzyme activity assay. Our both omics data sets on RBCs showed that glycolysis input was decreased, the PPP products and metabolism were elevated, and the oxidative reduction metabolites for antioxidation reactions were remarkably increased by gossypol treatment. Both metabolomics and proteomics data revealed a good causal correlation between enzymes involved in the specific pathways and the concordant alteration of metabolome. Through the global multi-omics analysis, we conclude that both glycolysis and PPP pathways responsible for essential energy metabolism and maintenance of reducing agents, along with the oxidative reduction pathways for antioxidation reactions, were involved in RBC in response to the hemolytic toxicity of gossypol. Our results may benefit further studies for developing gossypol as an anti-cancer drug.

## MATERIALS AND METHODS

### Chemicals and reagents

Gossypol-acetic acid (98% purity) was provided by Yangling Ciyuan Biotechnology Co., Ltd (Shanxi, China). Methanol, formic acid, acetonitrile, chloroform, and other chemicals were of high-performance liquid chromatography grade. Amino acid internal standard, methoxyamine hydrochloride, pyridine, triethylammonium bicarbonate, and iodoacetamide were purchased from Sigma-Aldrich (St. Louis, MO, USA). Bis (trimethylsilyl) trifluoroacetamide (BSTFA) and trimethylchlorosilane (TMCS) were obtained from Regis Technologies (Morton Grove, IL, USA). Dithiothreitol was obtained from Affymetrix (Santa Clara, CA).

### Animal treatment, sample collection and RBC parameters determination

Twelve gossypol-blank middle-lactating Holstein dairy cows were randomly divided into two groups: control group (four animals) and gossypol-treated group (eight animals). Animals in the gossypol-treatment group were administered with 29.58 ± 3.22 mg gossypol per kg body weight for 28 days, to reach 5-10 μM plasma negative gossypol, which shown to be effective inhibited cells proliferation *in vitro* study [4]. All animals were fed a gossypol-free diet and had *ad libitum* access to feed and water. Gossypol was transferred into capsules and administered to the animals twice a day. Animals in the control group were administered empty capsules.

On day 28, blood samples were collected from the jugular vein into EDTA-2K and sodium heparin-coated Vacutainer tubes (Greiner Bio-one GmbH, Kremsmünster, Austria). Blood anticoagulated by EDTA-2K were used for RBC parameters determination, and blood anticoagulated by sodium heparin were used for osmotic fragility determination and RBC separation. To separate RBC, plasma was removed by centrifugation at 1450*g* for 10 min. RBC pellets were washed with 0.9% saline, and the supernatant was removed by centrifugation at 1450*g* for 5 min. The wash process was repeated 3 times. The up layer leukocyte and platelet were discarded, and the lower layer RBCs were stored in liquid-nitrogen prior to analysis. The animal protocol was approved by the Animal Care and Use Committee of the Institute of Animal Science, Chinese Academy of Agricultural Sciences.

RBC parameters, including the amounts of RBC, HGB, mean corpuscular HGB and volume (MCH and MCV, respectively), and mean corpuscular HGB concentration (MCHC) were determined using a Mindray BC-2800Vet auto hematology analyzer (Shenzhen Mindray Bio-Medical Electronics Co., Ltd., Shenzhen, China). Osmotic fragility determination was performed according to a previously described method [33] by measuring the percentage of hemolyzed RBC in a solution of 0.8% NaCl-phosphate buffer, which was compared with that observed in distilled water (100% hemolysis).

### Metabolomics analysis

A 50-mg sample of RBC and 950 μL of methanol/chloroform/water solvent (volumetric ratio: 700:200:50) were successively added to an Eppendorf tube. The cell mixture was sonicated for 1 min on ice and incubated at −20°C for 1 min. This process was repeated five times. The mixture was then stored at −40°C for 24 h prior to centrifugation at 16,000*g* for 15 min at 4°C, followed by addition of 250 μL of the mixture to a GC vial containing 10 μL of internal standards (0.05 mg/mL of 13C3-15N-L-alanine, 13C5-15N-L-valine, 13C6-15N-L-leucine, and 13C6-15N-L-isoleucine). The mixture was dried under a gentle nitrogen stream, and 30 μL of 20 mg/mL methoxyamine hydrochloride in pyridine was added to the vial with the dry residue. The resulting mixture was vortexed vigorously for 30 s and incubated at 37°C for 90 min. BSTFA (30 μL, with 1% TMCS) was added to the mixture and derivatized at 70°C for 60 min prior to performing GC-MS metabolomics analysis. For UPLC-QTOF analysis, samples were sonicated as the same method described above for the GC-MS analysis. Then, the mixture was centrifuged at 16,000*g* for 10 min at 4°C. The supernatant (300 μL) was transferred to new tubes and dried under nitrogen gas, and the residue was resuspended in 100 μL acetonitrile/water (volumetric ratio: 50:50) solution for UPLC-QTOF analysis.

Metabolomics analysis was performed using an Agilent 7890A GC system coupled to an Agilent 5975C inert MSD system (Agilent Technologies, Santa Clara, CA, USA). A HP-5ms fused-silica capillary column (30 m × 0.25 mm × 0.25μm; Agilent J&W Scientific, Folsom, CA, USA) was used to separate the derivatives. Helium was used as a carrier gas at a constant flow rate of 1 mL/min. Injection volume was 1 μL in splitless mode, and the solvent-delay time was 6 min. The initial oven temperature was held at 70°C for 2 min, ramped to 160°C at a rate of 6 °C/min, then to 240°C at a rate of 10°C/min, to 300°C at a rate of 20°C/min, and finally held at 300°C for 6 min. The temperatures of the injector, transfer line, and electron-impact ion source were set to 250°C, 290°C, and 230°C, respectively. The impact energy was 70 eV, and data were collected in full-scan mode (m/z 50–600). AMDIS software was used to deconvolute mass spectra from raw GC-MS data, and the mass spectra were automatically matched using an in-house standard library, including retention time and mass spectra, the Golm Metabolome Database (http://gmd.mpimp-golm.mpg.de/), and the Agilent Fiehn GC/MS metabolomics RTL library (Agilent Technologies). Peak picking, alignment, deconvolution, and further processing of raw GC-MS data were performed according to previously described methods [34].

A 1290 series UPLC (Agilent Technologies) was used for separation on a ACQUITY UPLC HSS T3 columns (100 mm × i.d. 2.1 mm) with 1.7-μm particles at 25°C (Waters Corporation, Milford, MA, USA). The mobile phase consisted of formic acid/water (0.1%, solution A) and formic acid/acetonitrile (0.1%, solution B), and the flow rate was set at 0.3 mL/min. A gradient elution condition was applied as follows: 0-1.5 min, 1% B; 13 min, 99% B; 16.5 min, 99% B; 16.6 min, 1% B maintain to 20 min. MS data were collected using a TripleTOF 6600 (AB Sciex, concord, ON, Canada). Electrospray ionization was used as the ionization source, and analysis was performed in positive mode with a source temperature of 300°C. The scan was performed in MS1 range from 60 Da to 1000 Da. Parent ions were selected automatically using the IDA model in MS2. Collision energy was set at 35±15 ev. Metabolites according to UPLC-QTOF were identified by matching the MS1 and MS2 data in Lipidblast [35]. UPLC-QTOF raw data were transferred into mzXML format using the MSConvert software from Proteowizard [36]. Deconvoluted MS1 data (parent ions) and corresponding MS2 data (daughter ions) were obtained after peak matching, alignment, and retention-time correction using XCMS data-analysis software [37]. Isotope peaks and adduct ions were analyzed using the CAMERA package, and data were normalized against total peak intensities before performing univariate and multivariate statistical analysis.

### Sample preparation for proteomic analysis

RBC proteins were obtained by homogenizing 100 μL RBC samples in 700 μL 50 mM triethylammonium bicarbonate buffer (pH 8.5) containing 6 M urea. The homogenate was centrifuged at 2000 *g* for 15 min. Protein concentration were quantified by the BSA method, and checked by SDS-PAGE. An aliquot (100 μg) proteins was reduced in 100 μL system containing 50 mM dithiothreitol at 37°C for an hour. Cysteine residues were blocked with 10 μL of 1 M iodoacetamide for 30 min in the dark. The proteins were precipitated by acetone overnight and then digested using a trypsin (V511A, Promega) to substrate ratio of 50:1, at 37 °C overnight. The digested peptides were labeled with iTRAQ 8-plex reagents following the manufacturer’s instructions using 113-tags, 114-tag, 115-tag, and 116-tag, for control group, and 117-tag, 118-tag, 119-tag, and 121-tag, for gossypol-treated group, respectively. After labeling efficiency assessment, the eight samples were combined and subjected to high pH reverse phase fractionation.

The high reverse phase chromatography was carried out using Agilent 1290 high performance LC system with an autosampler and UV detection. The pooled and labeled peptides were loaded onto a C18 column (5 μm, 4.6×250 mm, Beijing TechMate Technology CO., LTD.) with 5 mM ammonium formate, 2% acetonitrile in water as buffer A (pH 9.0) and 5 mM ammonium formate, 10% water in acetonitrile as buffer B (pH 9.0). The LC was performed using a gradient from 5 to 80% of buffer B in 50 min at a flow rate of 0.6 mL/min. Forty fractions were collected at 1 min intervals and pooled into a total of 8 fractions, based on UV absorbance at 214 nm and with a multiple fraction concatenation strategy. The 8 pooled fractions were dried for subsequent nanoLC-MS/MS analysis.

### Protein identification and quantitation

Peptide fractions were reconstituted in 20 μL of 0.1% formic acid, 2% acetonitrile in water and analyzed by TripleTOF 6600 (Sciex, Framingham, MA, USA). Peptide mixtures were injected into the capillary column (0.075×150 mm) and separated by a 3 μm C18 column. Peptides were eluted with a liner gradient of 5-40% buffer B (0.1% formic acid, 2% water, and 5% DMSO in acetonitrile) at a flow rate of 0.3 μL for 90 min. Buffer A consisted of 0.1% formic acid, 2% acetonitrile, 5% DMSO (dissolved in acetonitrile) in water. The full MS1 scans were performed at the range of 350-1,500 m/z in 250 ms. The 40 most abundant ions with multiple charge states were selected for higher energy collision and the scan range was between 100-1500 m/z. The MS/MS spectra were obtained and searched using MaxQuant (version 2.5.6.5, Martinsried, Germany) against Uniprot *Bos taurus* protein database containing 6,887 sequences. Mass tolerances for precursor ions and fragment ions were 0.1 Da and 40 ppm, respectively. The proteins and peptides were filtered with a false discovery rate (FDR) < 1%. The enzyme parameter was limited to semi-tryptic peptides with a maximum miscleavage of 2. Variable modification of oxidation of methionine residue was selected, and carbamidomethylated cysteine, iTRAQ 8-plex (K) and iTRAQ 8-plex (N-term) were set as static modification.

### Enzyme activity assay

To confirm the results of proteomics analysis, the activities of four enzymes including 6-phosphogluconate dehydrogenase, flavin reductase (NADPH), glutathione S-transferase, and glutathione reductase which involved in the pathway of interests, were all determined by commercial kits purchased from Sigma-Aldrich (St. Louis, MO, USA). In the 6-phosphogluconate dehydrogenase kit, glucose-6-phosphate is oxidized to generate a product, which is specifically detected by colorimetric (450 nm) assay. NADPH was determined by measuring a fluorescent product (λ_ex_ = 535/λ_em_ = 587 nm) proportional to the amount of NADPH present. Glutathione S-transferase catalyzes the conjugation of L-glutathione to 1-Chloro-2, 4-dinitrobenzene through the thiol group of the glutathione. The reaction product absorbs at 340 nm. The rate of increase in the absorption is directly proportional to the glutathione S-transferase activity in the sample. The activity of glutathione reductase was measured by the decrease in absorbance caused by the oxidation of NADPH at 340 nm (UV assay).

### Experimental design and statistical rationale

The samples include four biological replicates for control group (n = 4) and eight biological replicates for gossypol-treated group (n = 8). Metabolome and proteome analyses were performed in randomized order to eliminate systematic biases. The normalized dataset was imported into MetaboAnalyst 3.0 [22] for principal component analysis (PCA) and partial least squares discriminant analysis (PLS-DA). Data from UPLC-QTOF were interquantile range filtered. All data were auto scaled prior to multivariate analysis. Univariate analysis was performed to screen potential differential metabolites and proteins. Student’s *t* test was conducted to compare the differential metabolites between the two groups. A fold change (FC), defined as normalized peak area of metabolites in treatment group/control group, was calculated as one of the options for differential metabolite selection. For the iTRAQ-based proteomics data, abundance of proteins was compared bases on Student’s *t* test carried out on log2-transferred iTRAQ ratio. Attention should be paid to the criteria selected for the differential metabolites and proteins. Compared with living organisms and other cells containing organelles, mature RBC do not possess a strong ability to resist stress due to the lack of all organelles, if the alteration is out of its endurance, hemolysis happens. Besides, the major aim of the metabolomics analysis was to screen pathways of change, which were further cross-validated by iTRAQ-based proteomics data set and confirmed by targeted-based enzymatic assays. On the basis of above, the following criteria were used for differential metabolites selection: *p* < 0.05, FC > 1.4 or < 0.67 (inter error was defined as the value of FC at which 90% of all metabolites had no deviation from each other). Metabolites exhibiting changes between the control and gossypol-treated groups were selected for pathway analysis using MetaboAnalyst 3.0 [22]. And differential abundance of proteins and enzymes activities with *p* < 0.1 with what ratio cutoff were considered when integrating with metabolomics results.

## SUPPLEMENTARY MATERIALS TABLES









## Abbreviations

RBC: red blood cell
HGB: hemoglobin
MCH: mean corpuscular hemoglobin
MCV: mean corpuscular volume
MCHC: mean corpuscular hemoglobin concentration
PCA: principal component analysis
PLS-DA: partial least squares discriminant analysis
FC: fold change
RSD: relative standard derivations
PPP: pentose phosphate pathway
