# Supplementary material for: Multi-omics analyses of red blood cell reveal antioxidation mechanisms associated with hemolytic toxicity of gossypol
Source: Oncotarget. 2017 Oct 10;8(61):103693–709. doi: 10.18632/oncotarget.21779 (PMC5732760; doi:10.18632/oncotarget.21779)
Supplement: Supplementary file 1 [file oncotarget-08-103693-s001.pdf]

Multi-omics analyses of red blood cell reveal antioxidation mechanisms associated with hemolytic toxicity of gossypol

SUPPLEMENTARY MATERIALS

Supplementary Materials 1: Source dataset of GC-MS metabolomics analysis

See Supplementary File 1

Supplementary Materials 2: Source dataset of UPLC-QTOF metabolomics analysis

See Supplementary File 2

Supplementary Materials 3: Source dataset of iTRAQ proteomics analysis

See Supplementary File 3
